# LncRNA SNHG1 alleviates IL-1β-induced osteoarthritis by inhibiting miR-16-5p-mediated p38 MAPK and NF-κB signaling pathways

**DOI:** 10.1042/BSR20191523

**Published:** 2019-09-06

**Authors:** Jinlai Lei, Yahui Fu, Yan Zhuang, Kun Zhang, Daigang Lu

**Affiliations:** Department of Orthopaedic Trauma, Honghui Hospital, Xi’an Jiaotong University, Xi’an, Shaanxi province 710054, China

**Keywords:** miR-16-5p, NF-κB, Osteoarthritis, p38MAPK, SNHG1

## Abstract

Long non-coding RNA (LncRNA) small nucleolar RNA host gene 1 (SNHG1) has been reported in the occurrence and development of several diseases, but its biological role and mechanism in osteoarthritis (OA) remain to be illuminated. In the present research, we aimed to investigate the effect of SNHG1 on IL-1β-induced OA and its molecular mechanism. Results revealed that SNHG1 decreased the expression of MMPs, ADAMTs, collagen, and aggrecan, and ameliorates IL-1β-induced metabolic dysfunction in normal human chondrocytes-keen. In addition, SNHG1 inhibited the expressions of pro-inflammatory cytokines in chondrocytes, including NO, PGE2, IL-6, TNF-α, i-NOS, and COX-2. Furthermore, luciferase reporter assay demonstrated that SNHG1 could directly interact with miR-16-5p and suppressed miR-16-5p expression and activity. What is more, miR-16-5p overexpression reversed SNHG1-inhibited aberrant catabolism and inflammation triggered by IL-1β stimulation. Finally, SNHG1 inhibits the expression of miR-16-5p-mediated factors involved in p38MAPK and NF-κB signaling pathways, including ERK1/2, p-p38 and p-p65. Taken together, the results of our studies illuminate that SNHG1 alleviates the inflammation of IL-1β-induced OA through the activation of miR-16-5p-mediated p38MAPK and NF-κB signaling pathway. It suggested that SNHG1 may serve as a potential target for OA diagnosis and treatment.

## Introduction

Osteoarthritis (OA) is a joint disease characterized by abnormal apoptosis of articular chondrocyte, cartilage sclerosis, and inflammation [1]. According to statistics, 10% of men and 13% of women aged 60 or older developed knee OA in the United States, and the number of OA patients continues to increase as the population ages and obesity increases [2]. OA is a heterogeneous disease that develops under the influence of a variety of factors. It is reported that many factors play a key role in the pathogenesis of OA such as IL-6, TNF-α, and IL-1β, and several inflammatory signaling pathways are involved in the regulation of OA [3].

NF-κB and p38MAPK, as critical inflammatory signaling pathways, play an important role in the development of OA. Studies have found that NF-κB and p38MAPK signaling pathway are activated in the articular cartilage and synovial cells of occurrence of OA [4]. In addition, the high expression of p-p38 may promote the expression of NF-κB, which plays an important role in the occurrence and development of OA [5]. Recent studies have reported the key role of NF-κB in joint health, which regulates the response to joint injury and inflammation by regulating cytokines, such as IL-1β, TNF-α [6]. It is well known that phosphorylation of p65 is essential in the NF-κB pathway, and Rap1, a critical protein, participates in the regulation of the NF-κB pathway by regulating the phosphorylation of p65 under physiological conditions such as inflammation or tissue repair [7]. Furthermore, it is reported that p38MAPK induces OA by mediating IL-1-induced down-regulation of aggregation proteoglycan gene expression in human chondrocytes [8].

The development of whole-genome sequencing technology showed that more than 95% of human genomes are dynamically transcribed. However, only 3% of transcripts encode proteins, and most transcripts are non-coding RNAs (ncRNAs) [9]. In these non-coding transcripts, long non-coding RNA (lncRNA) has attracted more and more attention. LncRNA is involved in a variety of biological processes, such as cell proliferation, differentiation, apoptosis, and inflammatory response [10,11]. Small nucleolar RNA host gene 1 (SNHG1) is a new type of lncRNA located at chromosome 11q12.3. More and more evidences showed that the dysfunction of SNHG1 is related to the occurrence and development of human diseases, such as OA, osteosarcoma, colorectal carcinoma, and liver cancer [12,13]. Recently, it has been reported that SNHG1 regulated gene expression through binding to some miRNAs in some diseases [14,15]. Zhang et al. reported that SNHG1 suppressed the proliferation, invasion, and metastasis of hepatocellular carcinoma cells by inhibiting the expression of miR-195 [16]. In addition, it is reported that SNHG1 regulates the differentiation of Treg cells and affects the immune escape of breast cancer by regulating miR-448/IDO pathways [17]. Besides, Chen et al. suggested that SNHG1 regulates the growth of colorectal cancer cells by interacting with miR-154-5p [18]. However, the mechanism of SNHG1 in OA has not been reported, and need to be further explored.

In the present study, we explored the role of SNHG1 in IL-1β-induced OA and its potential mechanisms. SNHG1 inhibited miR-16-5p-mediated p38 MAPK and NF-κB signaling pathways. The results provide a new insight that SNHG1 and miR-16-5p were significant indicators to detect the effect on OA diagnosis and treatment.

## Materials and methods

### Cell lines

The normal human articular chondrocytes-knee cells were obtained from Lonza Walkersville, Inc. (Walkersville American). The cells were cultured in DMEM Medium containing 10% FBS. Cells were maintained in a humidified incubator of 5% CO_2_ at 37°C.

### Reagents

IL-1β was obtained from T&L Biotechnology Co., LTD. (Beijing, China). DMEM medium and FBS were obtained from Genetime Biotechnology Co., Ltd. (Shanghai, China). Primescript™ reverse transcription kit and SYBR^®^ Premix Ex Taq™ II were purchased from TransGen Biotech (Beijing, China). Antibodies including anti-MMP-1, anti-MMP-3, anti-MMP-9, anti-ADAMS-4, anti-ADAMS-5, anti-collagen, anti-aggrecan, anti-iNOS, anti-COX-2, anti-p-p38, anti-p-ERK1/2, and anti-p-p65 were all obtained from Bioss Biotechnology Co., Ltd. (Beijing, China). GAPDH was purchased from Abcam Inc. (Cambridge, U.K.). The sequences of primers used in the present study was obtained from Tsingke (Beijing, China). ELISA kits were purchased from MSKBIO Technology Ltd. (Wuhan, China).

### Plasmids construction and cell transfection

Lnc-SNHG1 was synthesized and cloned into the expression vector pcDNA3.0 (GeneCreat, China). The plasmid vectors were transfected into human articular chondrocytes-knee cells using Lipofectamine2000 (Invitrogen, U.S.A.) according to the manufacturer’s instructions. The cells were incubated for 24 h before used in following assays.

### ELISA analysis

Normal human articular chondrocytes-knee cells was collected and processed according to the manufacturer’s protocols of the ELISA kit. The grinding fluid or standard was added to the plate, which was subsequently placed at 37°C for 40 min following mixing. Primary antibody working fluid, enzyme conjugate, and TMB solution were added sequentially subsequent to wash the plate. The absorbance was measured at 450 nm using a microplate reader.

### Gelatin zymography assays of MMP-9 activity

MMP-9 activity was determined by gelatin zymography assays as previously described. Briefly, The samples mixed with SDS sample buffer were subjected to 10% SDS/PAGE gels polymerized with 0.1% gelatin. The gel was then washed in 2.5% Triton X-100 and incubated in radioimmunoprecipitation buffer (Nanjing KeyGen Biotech Co., Ltd.) at 37°C for 12 h. The gel was stained with 0.05% Coomassie brilliant blue stain R-250 (Beyotime Institute of Biotechnology) for visualization.

### Luciferase reporter assay

For luciferase reporter analysis of NF-κB promoter, the core promoters of the NF-κB gene were synthesized and cloned into the pGL3 alkaline firefly luciferase reporter (Transheep, China). The pRL-TK vector was used as control. For luciferase reporter analysis of miR-16-5p target gene, target sequences containing predicted binding sites of miR-16-5p were synthesized and inserted into pmirGLO luciferase vector (Transheep, China). Luciferase activity was determined by Dual Luciferase Assay system (Promega, U.S.A.). The activity of luciferase was standardized as firefly luciferase.

### RNA immunoprecipitation assay

The EZ Magna RNA immunoprecipitation Kit (Genecreate, China) was used according to the manufacturer’s protocol. Briefly, normal human articular chondrocytes-knee cells were lysed in RIP lysis buffer. Magnetic beads were incubated with antibodies for 1 h, and then cell lysates was immunoprecipitated with beads for 8 h at 4°C at room temperature. Finally, RNA was purified and measured by RT-qPCR.

### RT-qPCR

The total RNA was extracted from cells using TRIzol reagent (Invitrogen, Carlsbad, CA, U.S.A.) following to the guidelines. Reverse transcription for mRNAs were performed using a Revert Aid™ First Strand cDNA Synthesis kit (TaKaRa, Shiga, Japan), according to the manufacturer’s instructions. The thermocycling conditions consisted of: 60 s at 95°C, followed by 32 cycles (15 s at 95°C, 20 s at 60°C, and 10 s at 72°C). The expression of mRNA was normalized to β-actin expression using the 2^−ΔΔ*C*^_T_ method [19]. GAPDH was used as internal control. Furthermore, the primers used in the present research are listed in Table 1.

**Table 1:** Primer sequences used in reverse transcription quantitative PCR

Gene	Primer sequence (5′→3′)
GAPDH	F: AAG GTG AAG GTC GGA GTC A
	R: GGA AGA TGG TGA TGG GAT TT
MMP-1	F: AGT GTG TCA CCA TAC CAA GCA C
	R: CGA GGC ACC AAG ACT GGA AGA
MMP-3	F: CAA GGG CCT CTT CTG CGA TTT CG
	R: CGG TAG GCA GCT AGG GCA GGG C
MMP-9	F: GTC CCA CCC ACC CCA GAC AGA
	R: GAG CCA CAA ACT GCA GGT GGT
ADAMTS-4	F: GAT CGA TGC CGG TGC TAA GA
	R: TCC TAT GGG AGA ACG GCA GA
ADAMTS-5	F: CTT CGG CAG CAC ATA TAC
	R: GAA CGC TTC ACG AAT TTG C
collagen Ⅱ	F: TGC AGG CTG GAG ATC CTA CT
	R: TTC TAG ACG GCA GGT CAG GT
aggrecan	F: AGG CTG AAG TTA CAG GTC
	R: TTG GCT CCC AGT GTC TTA
miR-16-5p	F: CAG TGC GTG TCG TGG AGT
	R: AGG TCC AGT TTT CCC AGG
SNHG1	F: AGG CTG AAG TTA CAG GTC
	R: TTG GCT CCC AGT GTC TTA

### Western blot analysis

Samples were treated with protein extraction reagent and centrifuged at 10,000×***g*** at 4°C for 20 min to obtain the supernatant according to the manufacturer’s protocols. The lysates were separated on SDS-PAGE (12%) and transferred onto a PVDF membranes. The blots were blocked with fresh 5% nonfat milk for 2 h at room temperature and then incubated with the primary antibodies against MMP-1 (1:500), MMP-3 (1:800), MMP-9 (1:500), ADAMS-4 (1:500), ADAMS-5 (1:500), collagen (1:1000), aggrecan (1:800), iNOS (1:1500), COX-2 (1:800), p-p38 (1:1000), and ERK1/2 (1:500), p-p65 (1:800). Following three washes with TBST and incubated with secondary antibodies for 1 h. Bands were visualized with an enhanced chemiluminescent substrate kit (Amersham Pharmacia). Densitometric measurements were performed using ImageJ computer software.

### Statistical analyses

All experiments were repeated three times. Data are presented as the mean ± standard deviation. All statistical analysis were carried out using SPSS 20.0, (Chicago, IL, U.S.A.). All experiments were analyzed by the Student’s *t* test. A value of *P*<0.05 was considered statistically significant.

## Results

### Overexpression of SNHG1 ameliorates IL-1β-induced metabolic dysfunction in chondrocytes

**Figure 1:**
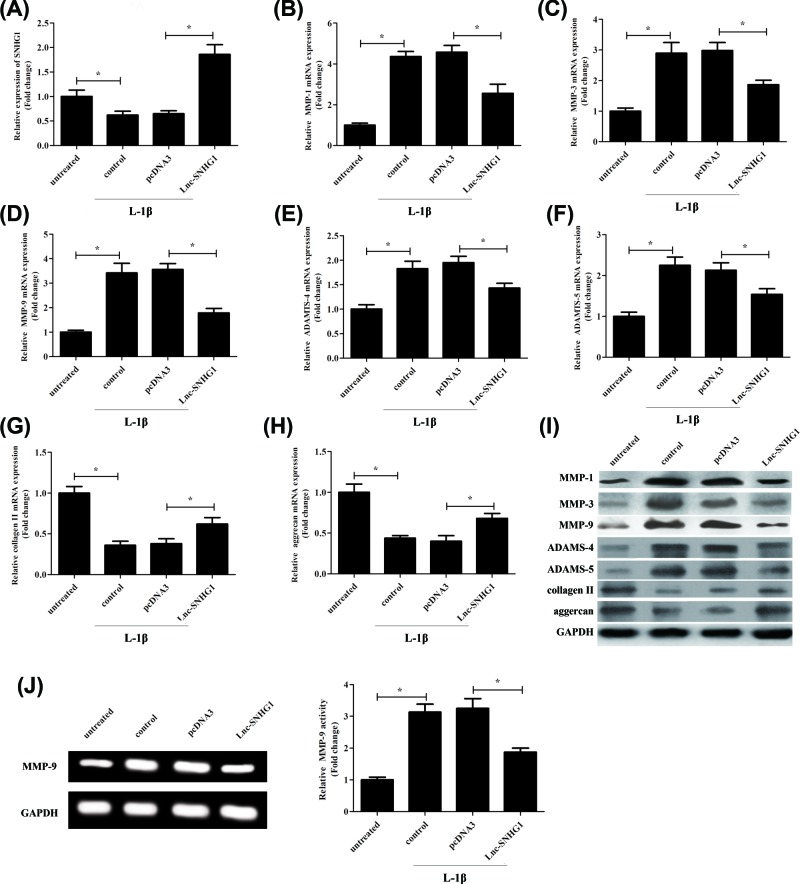
Overexpression of SNHG1 ameliorates IL-1β-induced metabolic dysfunction in chondrocytes Normal human articular chondrocytes-keen were transfected with empty plasmid pcDNA3.0 or overexpression plasmid lnc-SNHG1, respectively and then treated with IL-1β (10 ng/ml) for 24 h. (**A**) RT-qPCR was used to confirm the expression levels of SNHG1. (**B–H**) RT-qPCR was used to detect the expression levels of MMP-1, MMP-3, MMP-3, ADAMTS-4, ADAMTS-5, collagen II, and aggrecan. (**I**) Western blot was performed to detect the protein expression levels of MMP-1, MMP-3, MMP-9, ADAMTS-4, ADAMTS-5, collagen II, and aggrecan. (**J**) The activity of MMO-9 was determined by Gelatin zymography assay. ‘*’ means *P*<0.05. GAPHD was used as an invariant internal control for calculating protein-fold changes.

Normal human chondrocytes-keen were transfected with empty plasmid pcDNA3.0 or overexpression plasmid lnc-SNHG1, respectively and then treated with IL-1β (10 ng/ml) for 24 h. The SNHG1 expression was measured by RT-qPCR. Results exhibited that the SNHG1 expression was decreased after treatment with IL-1β, and SNHG1 was most highly expressed in lnc-SNHG1 group compared with pcDNA3 group, while there was no significant difference in the expression of SNHG1 in control group and pcDNA3 group (Figure 1A). RT-qPCR was used to detect the expression levels of MMP-1, MMP-3, MMP-9, ADAMTS-4, ADAMTS-5, collagen II, and aggrecan. As expected, the expression of MMP-1, MMP-3, MMP-9, ADAMTS-4, and ADAMTS-5 was decreased significantly when transfected with lnc-SNHG1 compared with pcDNA3 (Figure 1B–F), while the expression of collagen II and aggrecan was increased significantly in lnc-SNHG1 group (Figure 1G,H). Moreover, the results were further validated by western blot at protein level. The results were consistent with the level of mRNA (Figure 1I). Besides, lnc-SNHG1 transfection significantly promoted gelatinolytic activity of MMP-9 (Figure 1J).

### Negative regulation of the IL-1β-stimulated production of proinflammatory cytokines in human chondrocytes by SNHG1

**Figure 2:**
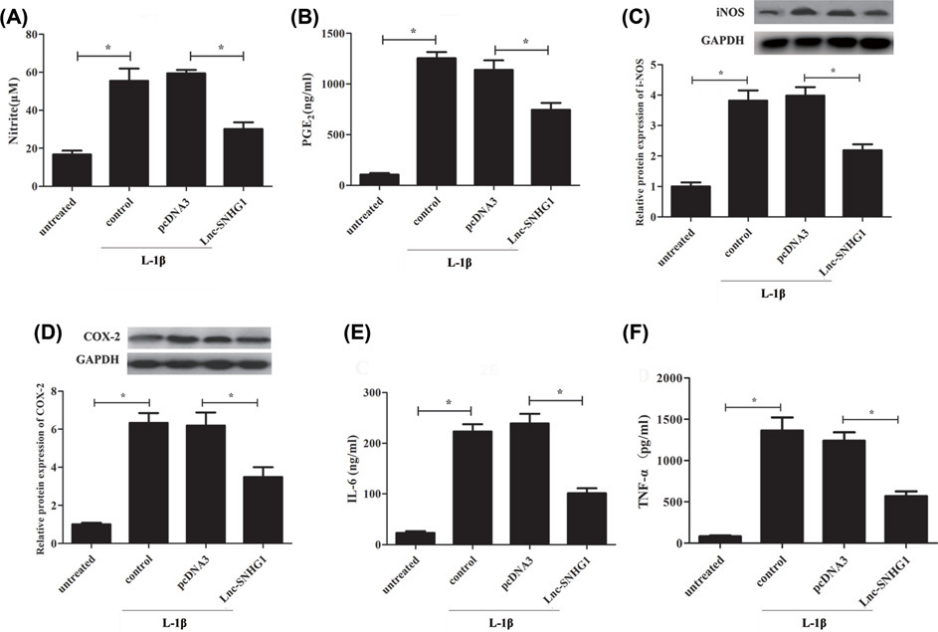
Negative regulation of the IL-1β-stimulated production of proinflammatory cytokines in human chondrocytes by SNHG1 Normal human articular chondrocytes-keen were transfected with empty plasmid pcDNA3.0 or overexpression plasmid lnc-SNHG1, respectively and then treated with IL-1β (10 ng/ml) for 24 h. (**A,B,E,F**) ELISA was performed to measure the levels of NO, PGE2, IL-6, and TNF-α. (**C,D**) Western blot was used to confirm the protein expression levels of i-NOS and COX-2. ‘*’ means *P*<0.05. GAPHD was used as an invariant internal control for calculating protein-fold changes.

ELISA was performed to measure the levels of several pro-inflammatory cytokines, including NO, PGE2, IL-6, and TNF-α. Results showed that the levels of these cytokines were decreased significantly in lnc-SNHG1 group compared with pcDNA3 group (Figure 2A,B,E,F). Furthermore, western blot was used to confirm the protein expression levels of i-NOS and COX-2. Results exhibited that the protein expression of i-NOS and COX-2 was decreased significantly when transfected with lnc-SNHG1 compared with pcDNA3 in human articular chondrocytes. (Figure 2C,D).

### SNHG1 regulates miR-16-5p expression and activity

**Figure 3:**
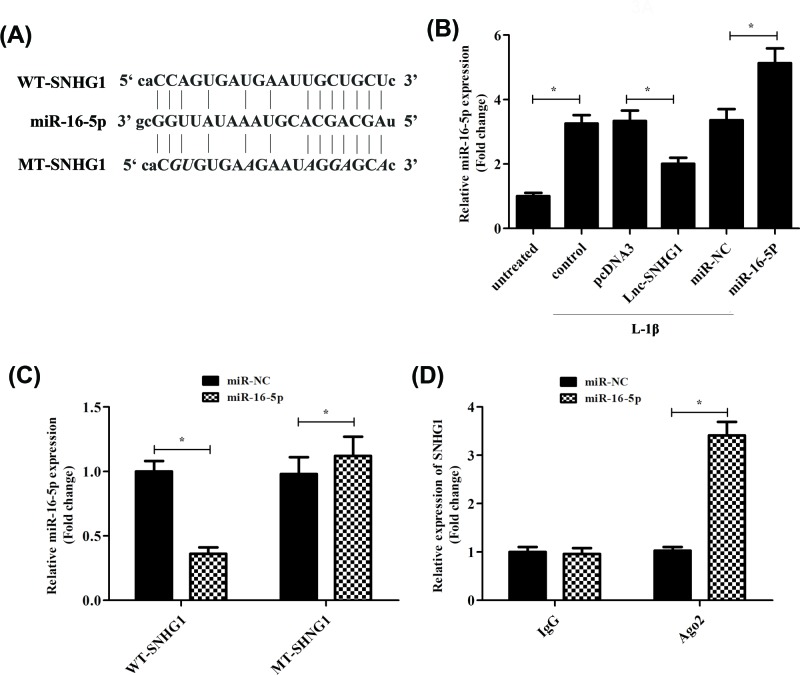
SNHG1 regulates miR-16-5p expression and activity (**A**) Sequence of the putative or mutated SNHG1 binding site within the 3′-UTR of miR-16-5p. Normal human articular chondrocytes-keen were transfected with empty plasmid pcDNA3.0, lnc-SNHG1, miR-NC, or miR-16-5p, respectively and then treated with IL-1β (10 ng/ml) for 24 h. (**B**) RT-qPCR was used to confirm the expression levels of miR-16-5p. (**C**) Dual luciferase reporter assay was conducted with wild-type and mutant-type (putative binding sites for miR-154-5p were mutated) luciferase reporter vectors, and then confirmed the levels of luciferase activity. (**D**) RIP with an anti-Ago2 antibody was used to assess endogenous Ago2 binding to RNA, and IgG was used as the control. SNHG1 levels were determined by RT-qPCR and presented as fold enrichment in Ago2 relative to input. ‘*’ means *P*<0.05, and ‘**’ means *P*<0.01. GAPHD was used as an invariant internal control for calculating protein-fold changes.

In order to investigate the potential mechanism by which SNHG1 in human chondrocyte malignant phenotype, we decided to search for the downstream target gene of SNHG1. Many cytoplasmic lncRNAs have been reported to function as competitive endogenous RNAs via competitive binding to miRNA. So, we first used miRDB database blast to predict the target miRNA of SNHG1. We found that SNHG1 had a putative miR-16-5p targetting site (Figure 3A). Luciferase reporter assay was performed to determine whether miR-16-5p could directly interact with SNHG1 in human articular chondrocytes. We constructed wild-type and mutant-type (putative binding sites for miR-16-5p were mutated) SNHG1 luciferase reporter vectors. As shown in Figure 3C, co-transfection of the wild-type SNHG1 vector (WT-SNHG1) with miR-16-5p mimic, significantly reduced luciferase activities compared with MT-SNHG1 (Figure 3C). Furthermore, RT-qPCR was used to measure the expression levels of miR-16-5p when the cells were transfection with pcDNA3.0, lnc-SNHG1, miR-NC, or miR-16-5p respectively. As expected, the expression of miR-16-5p was decreased significantly when transfected with lnc-SNHG1 compared with pcDNA3. It is further proved that miR-16-5p is the target miRNA of SNHG1. Besides, the expression of miR-16-5p was increased significantly when transfected with miR-16-5p compared with miR-NC (Figure 3B). In addition, RNA immunoprecipitation assay results revealed that SNHG1 was significantly enriched in the miR-16-5p complex containing Ago2 used the specific primer of SNHG1 compared with the control primers, which indicated that SNHG1 and miR16-5p were directly bound in human articular chondrocytes (Figure 3D).

### miR-16-5p overexpression reversed SNHG1-inhibited aberrant catabolism and inflammation triggered by IL-1β stimulation

**Figure 4:**
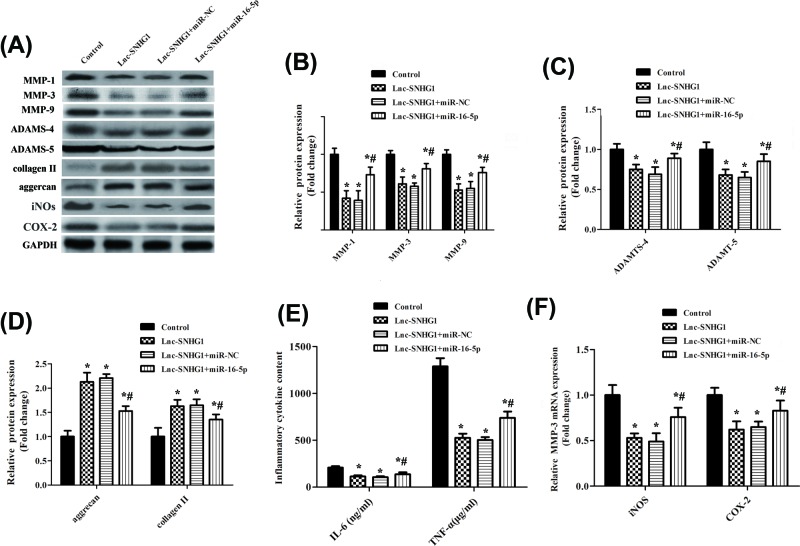
miR-16-5p overexpression reversed SNHG1-inhibited aberrant catabolism and inflammation triggered by IL-1β stimulation Normal human articular chondrocytes-keen were transfected with lnc-SNHG1, lnc-SNHG1+ miR-NC, or lnc-SNHG1+ miR-16-5p, respectively and then treated with IL-1β (10 ng/ml) for 24 h. (**A**) Western blot was performed to detect the protein expression levels of MMP-1, MMP-3, MMP-9, ADAMTS-4, ADAMTS-5, aggrecan, collagen II, i-NOS, and COX-2. (**B,C,D,F**) Quantitation of band intensity in (A). (**E**) ELISA was performed to measure the levels of IL-6 and TNF-α. ‘*’ means compared with control group *P*<0.05, and ‘#’ means compared with lnc-SNHG1or lnc-SNHG1+ miR-NC group *P*<0.05. GAPHD was used as an invariant internal control for calculating protein-fold changes.

We next examined the effect of miR-16-5p on catabolism and inflammation in chondrocytes. Western blot was performed to detect the expression of inflammatory and metabolic factors, including MMP-1, MMP-3, MMP-9, ADAMTS-4, ADAMTS-5, i-NOS, and COX-2 (figure 4A). Results confirmed that the protein expression of these factors was up-regulated significantly when co-transfection of miR-16-5p and lnc-SNHG1 compared with co-transfection of lnc-SNHG1and miR-NC, suggesting that miR-16-5p overexpression reversed SNHG1-inhibited aberrant catabolism and inflammation in chondrocytes (Figure 4B,C,F). While, the expression of collagen II and aggrecan was decreased significantly in lnc-SNHG1+ miR-16-5p group (Figure 4D). In addition, the IL-6 and TNF-α levels were increased significantly when co-transfection of miR-16-5p and lnc-SNHG1 compared with lnc-SNHG1+ miR-NC group and lnc-SNHG1 group (Figure 4E).

### SNHG1 inhibited the activation of miR-16-5p-mediated p38 MAPK and NF-κB signaling

**Figure 5:**
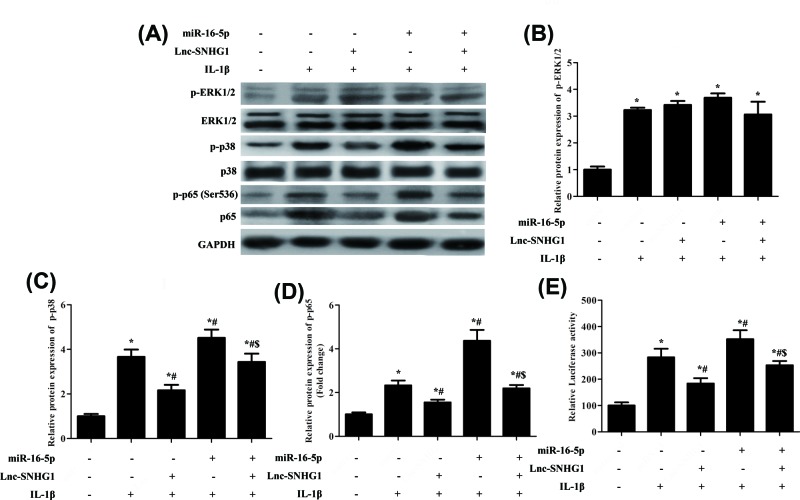
SNHG1 inhibited the activation of miR-16-5p-mediated p38 MAPK and NF-κB signaling Normal human articular chondrocytes-keen were transfected with lnc-SNHG1 or miR-16-5p, respectively and then treated with IL-1β (10 ng/ml) for 24 h. (**A**–**D**) Western blot was performed to confirm the protein expression levels of p-ERK1/2, ERK1/2, p-p38, p38, p-p65 and p65. (**E**) Dual luciferase reporter assay was performed to measure the activity of NF-κB. ‘*’ means compared with control group *P*<0.05, ‘#’ means compared with lnc-SNHG1 group *P*<0.05, and ‘$’ means compared with lnc-SNHG1or miR-16-5p group *P*<0.05. GAPHD was used as an invariant internal control for calculating protein-fold changes.

Studies demonstrated that some miRNA could act as an inflammation suppressor gene through targetting p38 MAPK and NF-κB signaling pathways [20]. Using expression analyses, we found that when SNHG1 was overexpressed, the protein expression levels of p-ERK1/2, p-p38, and p-p65 were down-regulated, while overexpression of miR-154-5p in SNHG1-down-regulated cells reversed the reduction. When co-transfection of miR-16-5p and lnc-SNHG1, the expression levels of p-ERK1/2, p-p38, and p-p65 were decreased significantly compared with transfection of miR-16-5p or lnc-SNHG1, respectively (Figure 5A–D), suggesting that SNHG1 inhibited the activation of miR-16-5p-mediated p38 MAPK and NF-κB signaling. Moreover, the result of luciferase reporter assays showed that NF-κB activity was increased when transfected with miR-16-5p compared with transfection of lnc-SNHG1and control group while when co-transfection of miR-16-5p and lnc-SNHG1 reversed the phenomenon (Figure 5E).

## Discussion

OA is a degenerative disease, which is caused by ageing, obesity, strain, trauma, inflammation, congenital abnormalities of joint, and joint deformity [21]. Earlier studies have shown that the occurrence and development of OA are associated with a variety of lncRNA. For example, in temporomandibular OA, up-regulation of lncRNA HOTAIR expression contributed to IL-1β-induced MMP overexpression and chondrocyte apoptosis [22]. In addition, lncRNA SNHG5 enhanced OA chondrocyte proliferation by regulating miR-26a/SOX2 signal axis [23]. Furthermore, Xu et al. reported that lncRNA MEG3 induced the occurrence and development of OA through down-regulation of miR-16/SMAD7 axis [24]. The purpose of the present study was to explore the effect and mechanism of SNHG1 on OA.

In recent years, it has been found that SNHG1 is involved in carcinogenesis of human cancer by activating different signaling pathways [14,15,25]. Zhang et al. indicated that SNHG1 enhances the proliferation and migration of prostate cancer cells by regulating the expression of miR-199a-3p/CDK7 axis [26]. Jiang et al. found that overexpression of SNHG1 promotes cell proliferation, migration, and EMT in OA. Moreover, miR-577 as a ceRNA of SNHG1 in osteosarcoma cells. In addition, WNT2B acts as a target of miR-577, and WNT2B plays a carcinogenic role by activating Wnt/β-catenin pathway in OS cells [27]. Besides, it is reported that SNHG1 increased the expression of human oncogene nin-one binding protein (nob1) by sponging miR-326 as ceRNA, and ultimately promoted the growth, migration, and invasion of osteosarcoma cells [28]. However, the relationship between SNHG1 and OA remains to be elucidated. In the present study, we found that SNHG1 could improve IL-1β-induced dysmetabolism of chondrocytes and reduce the expression of pro-inflammatory cytokines. These results suggest that SNHG1 inhibited the occurrence and development of OA. According to microRNA software (miRDB), miR-16-5p may be the direct target of SNHG1. Therefore, we speculate that SNHG1 may regulate the catabolism and inflammation through miR-16-5p in OA.

We found a negative correlation between the expression of SNHG1 and miR-16-5p in chondrocytes, and miR-16-5p overexpression reversed SNHG1-inhibited aberrant catabolism and inflammation. Next, we dedicated to figure out how SNHG1/miR-16-5p contribute to the catabolism and inflammation of OA. Zhang et al. reported that miR-16-5p expression considerably decreased with the severity of bone destruction and miR-16-5p mimics significantly reduced RANKL-induced osteoclast formation. However, miR-16-5p inhibitors can significantly promote osteoclast formation. These results suggest that miR-16-5p may be a therapeutic target for giant cell tumors of bone by inhibiting osteoclast genesis [29]. Besides, some researchers suggested that NF-κB and p38MAPK signaling pathway play a vital role in the inflammation of chondrocytes [30]*.* Johanne et al. indicated that NF-κB induced the production of NO, COX-2, and prostaglandin E2, promoted the catabolism of MMPs and promoted the production of MMPs and ADAMs, and ultimately led to the degradation of articular cartilage [31]*.* These reports inspired us to ascertain whether SNHG1/miR-16-5p exerted an important role in OA via the NF-κB and p38 MAPK signaling pathway. We have demonstrated that miR-16-5p promoted the expression levels of p-ERK1/2, p-p38, p-p65, while SNHG1 inhibited the expression of these genes which were mediated by miR-16-5p in NF-κB and p38 MAPK signaling. The results suggested that SNHG1 inhibits the development of OA through miR-16-5p-mediated NF-κB and p38 MAPK signaling pathways.

Inflammatory signaling underlies many diseases, from arthritis to cancer. NF-κB is one of the most important signaling pathways in inflammation. So far, our understanding and exploration of NF-κB have begun to understand the possible role of non-coding RNA (ncRNA), especially lncRNA [32]. It has been reported that lncRNA NKILA activates NF-κB signaling in MDA-MB-231 breast cancer cells by stimulating up-regulation, such as TNF-α or lipopolysaccharide (LPS) [33]. THRIL is another lncRNA in the NF-kB pathway and was first identified in the unbiased microarray screening of theTHP1 human macrophage cell lines flowing innate immune activation. Pull-down experiments showed that it could form complex with hnRNPL, which is essential for regulating the transcription of the gene encoding TNF-a [34]. Accordingly, the functional relevance of lncRNAs in the regulation of NF-κB effector proteins and inflammatory gene expression could signify that dysregulation of such lncRNAs has far-reaching implications in the pathogenesis of various, yet-to-be associated, chronic inflammatory conditions.

In conclusion, our current study reveals that lncRNA SNHG1 alleviates IL-1β-induced OA, and the specific mechanism was that SNHG1 inhibited miR-16-5p-mediated p38 MAPK and NF-κB signaling pathways. It provides a theoretical reference for the treatment of OA. It provides a new theoretical reference for the treatment of OA. Therefore, SNHG1 acts as a novel lncRNA and may serve as a potential therapeutic target in OA.

## Abbreviations

ADAMT: aggrecanases
GAPDH: glyceraldehyde-3-phosphate dehydrogenase
LncRNA: long non-coding RNA
MAPK: mitogen-activated protein kinase
MMP: matrix metalloproteinases
ncRNA: non-coding RNA
NO: nitric oxide
OA: osteoarthritis
OS: osteosarcoma
PGE2: prostaglandin E2
SNHG1: small nucleolar RNA host gene 1
